# Influence of Resident Education in Correctly Diagnosing Extremity Soft Tissue Sarcoma

**DOI:** 10.1155/2013/679323

**Published:** 2013-04-18

**Authors:** Vignesh K. Alamanda, Samuel N. Crosby, Shannon L. Mathis, Kristin R. Archer, Kyla P. Terhune, Ginger E. Holt

**Affiliations:** ^1^Department of Orthopaedics and Rehabilitation, Vanderbilt University Medical Center, 1215 21st Avenue South, Medical Center East, South Tower, Suite 4200, Nashville, TN 37232-8774, USA; ^2^Division of General Surgery, Vanderbilt University Medical Center, Nashville, TN 37232, USA

## Abstract

*Background*. One-third of all extremity soft tissue sarcomas are misdiagnosed and inappropriately excised without proper preoperative diagnosis and planning. This study aimed at examining the clinical judgment of residents in both general and orthopaedic surgery and at determining whether resident education plays a role in appropriately managing unknown soft tissue masses. *Methods*. A case-based survey was used to assess clinical decisions, practice patterns, and demographics. Aggregate response for all of the clinical cases by each respondent was correlated with the selections made for practice patterns and demographic data. *Results*. A total of 381 responses were returned. A higher percentage of respondents from the orthopaedic group (84.2%) noted having a dedicated STS rotation as compared to the general surgery group (35.8%) *P* < 0.001. Depth, size, and location of the mass, rate of growth, and imaging characteristics were considered to be important factors. Each additional year of training resulted in 10% increased odds of selecting the correct clinical decision for both groups. *Conclusion*. Our study showed that current residents in both orthopaedic surgery and general surgery are able to appropriately identify patients with suspicious masses. Continuing education in sarcoma care should be implemented beyond the years of residency training.

## 1. Introduction

Soft tissue sarcomas (STS) are highly malignant and rare tumors with an incidence of about 1 in every 100,000 patients [1]. In contrast, their benign counterparts are much more common with an incidence of about 300 in every 100,000 patients [2]. The relatively high incidence of benign soft tissue neoplasms as compared to malignant STS results in many incomplete excisions of STS without adequate preoperative planning, biopsy, and imaging. At our institution, approximately two-third of patients present for a primary resection while the remaining one-third present for a re-resection of an incompletely excised tumor. These unplanned excisions often leave positive surgical margins and necessitate a much larger repeat excision to obtain clear surgical margins. This subsequently results in greater emotional tolls, higher costs, and inferior functional outcomes [3].

While it is well established that STS are often mistakenly excised under the pretense of a benign tumor, it is not completely known how the physician's graduate medical education training plays a role in this. In an attempt to understand this further, we administered a case-based sarcoma survey that assessed the respondent is clinical decision making skills on the diagnosis of STS and their practice patterns. This allowed us to examine the association between different variables involved in the residents' education and general practice guidelines and the decisions that they chose for the clinical cases. This also allowed us to identify factors for improvement in surgical education in both orthopaedics and general surgery residency programs to reduce the incidence of inappropriate excisions as these residents progress to independent practice. 

## 2. Methods

Following consent from our home institution's review board, previous incomplete excisions that were referred to our institution for reexcision were analyzed as cases for this survey. A total of eight cases of incompletely excised STS were selected. Each selection included a clinical vignette with a brief clinical history and presenting symptoms along with appropriate images (either CT or MRI). Residents were asked to choose the next clinical step for each case. Options included “Mass is likely benign. Preoperative diagnosis is not crucial and mass can be excised” and “Mass is likely malignant. Preoperative diagnosis is crucial and mass needs a FNA/core needle biopsy.” The validity of the case scenarios was verified with senior faculty specifically trained in musculoskeletal oncology at our institution; the survey was subsequently distributed to the orthopaedic and general surgery residents at our home institution for pilot data. 

The national survey was designed using input gathered from the initial pilot survey. Additional questions such as the clinical criteria that were used to guide them to the decision were also assessed. Demographics and other information such as the number of years of residency training that they have completed were also assessed.

An email describing the purpose of the survey and the survey itself was electronically forwarded to all the program directors of residency programs of both general surgery and orthopaedic surgery with a request to forward it to their respective residents. Follow-up reminders were sent for a total of five times with two weeks between each request. Study data were collected and managed using REDCap electronic data capture tools hosted at our home institution [4]. REDCap (Research Electronic Data Capture) is a secure, web-based application designed to support data capture for research studies.

Descriptive techniques were used to determine the distribution of the two clinical steps and overall percent of correct answers was determined for each resident. Student's *t*-tests and chi-square or Fisher's exact tests were used to assess differences in demographics by type of residency program (general surgery versus orthopaedics). Wilcoxon Rank Sum tests examined differences in practice patterns and preferences by programs. Bivariate mixed model logistic regression analyses were conducted to examine the association between demographics, practice patterns, and preferences and the clinical step selected. A random intercept was included in all analyses to account for the clustering of responses by resident. Stata statistical software (Version 11.0; StataCorp., College Station, TX) was used to analyze the data. The significance level was set at *P* < 0.05.

## 3. Results

A total of 247 and 134 complete responses from the orthopaedic surgery and the general surgery group, respectively, were returned and analyzed (Table 1). Responses were obtained from residents in 84 different institutions for the orthopaedic surgery group and 77 different institutions for the general surgery group. The average age of respondents in both groups was 30.5-year old for the general surgery group and 30.1-year old for the orthopaedics group. Significant differences in the demographic characteristics between the two groups included gender differences. In the general surgery group, the gender split was 65% females and 35% males while in the orthopaedic surgery group, the split was 80% males and 20% females; *P* < 0.001. It was also noted that a significantly higher proportion of residents in orthopaedic surgery had a dedicated STS rotation where they saw and took care of patients with STS (84% in orthopaedics versus only 36% in general surgery). Among respondents who reported not having a dedicated STS rotation, 97% from the orthopaedics group reported having a dedicated STS teaching (versus 67% for the general surgery group). 

Analysis of the clinical vignettes revealed that on average the general surgery group had a correct response rate of 55.8% while the orthopaedic surgery group had a correct response rate of 56.4% (Table 2). The difference was insignificant with a *P* value of 0.81. The lowest correct response rate was 34.4% for the third vignette and the highest correct response rate was 75.4% for the fourth vignette. 

As part of the survey, residents were asked to rate the degree to which a variety of factors would increase their suspicion for malignancy (Table 3). Factors that the respondents were asked to rate included characteristics of depth of the mass, rate of growth of the mass, imaging characteristics, location and mobility of the mass, and age of the patient. Scoring for each of the factors was on a 1 to 6 scale, with 1 indicating “Strongly Disagree” and 6 indicating “Strongly Agree.” All factors were given a rating between 4.4 and 5.6 in both groups. Both groups considered the rate of growth of the mass and imaging characteristics as very important factors in deciding the malignancy of the mass. Other factors including the mobility of the mass and age of the patient were also considered as important (with average scores ranging from 4.4 to 4.6) factors. The average response to largest size tumor that the residents would be comfortable with excising without a biopsy was 3.8 cm in both groups. 

Similar to earlier parts of the survey, residents were also asked to rate factors that impeded their decision to perform a biopsy (Table 4). Factors were again rated from 1 to 6 with 1 indicating “Strongly Disagree” and 6 indicating “Strongly Agree.” The respondents most strongly agreed that an impediment in performing a biopsy was the assumption that the entire specimen would lead to a more accurate diagnosis (score of 3.5 and 3.7 by general surgery and orthopaedics, resp.). Concern about inconveniencing the pathologist was the least influential in their decision. Orthopaedic surgery residents in our survey group were more likely to consider soft tissue masses as being malignant than their general surgery counterparts (*P* < 0.001). The orthopaedics group was also less likely to be confident about pathology to make a diagnosis based on a biopsy specimen as compared to the general surgery group. Analysis also revealed that the respondents from the orthopaedics group felt that they were not extensively trained in the use of biopsies in residency or fellowship as compared to the general surgery group (*P* = 0.02). 

Bivariate analyses were performed for both the general surgery group and orthopaedics group to correlate selecting the correct clinical decision with the ability to rank the important clinical factors (Tables 5 and 6). In analyzing the results, the only factor associated with the respondents selecting the correct clinical decision was the years of training that they had completed (*P* = 0.04); for each year of training that the respondent completed, the chance of correctly identifying the next clinical decision increased by a factor of 1.11. 

When the respondents from the orthopaedics group were analyzed, years of training also played a role in increasing one's chances of selecting the appropriate clinical decision (*P* = 0.01). For every year of training that was completed, the odds of selecting the correct decision were increased by a factor of 1.10. Additional factors that played a role in identifying the correct response were also identified. For every 1 cm increase of tumor size that the respondents were comfortable excising without a biopsy, there is a 6% reduction in the odds of selecting the correct decision (*P* = 0.02). Similarly, for every one score increase that agree with the statement that the age of the patient is an important factor in raising suspicion for malignancy, there is 14% reduction in the odds of selecting the correct clinical decision (*P* < 0.006). For every one score increase that agree with the statement that they do not want to inconvenience the pathologist, there is a 16% reduction in the odds of selecting the correct clinical decision (*P* = 0.03).

## 4. Discussion

Soft tissue sarcomas are often misdiagnosed and excised under the assumption of being a benign tumor [2]. To understand how postgraduate education and training play a role in this misdiagnosis, residents from general surgery and orthopaedics were surveyed from across the United States for their practice patterns, demographics, and the clinical decision that they would make for sample clinical scenarios. 

Results from this study showed a significantly higher proportion of residents in orthopaedic surgery training programs (84%) as having a dedicated rotation in which they saw and took care of STS patients as compared to residents in general surgery programs (36%). Yet, results were similar when the vignette answers were analyzed for correct responses (55.8% for general surgery and 56.4% for orthopaedic surgery).

The only factor associated with residents selecting the correct clinical decision was the years of training that they had completed (*P* = 0.04); for each year of training that the respondent completed, the odds of correctly identifying the next step in clinical decision increased by a factor of 1.11. This can be explained in part by the accumulation of broad surgical knowledge and experience. One may argue that residents in both general and orthopaedic surgery identified the same number of sarcomas correctly, with general surgery programs in this survey reporting 30% less STS teaching rotations. We attribute this to standardized study practices for in-training exams and board certification examinations that are generally designed to teach safe surgical skills with standardized preparatory materials that emphasize minimizing errors in treating STS. 

Depth, location, rate of growth, and imaging characteristics of masses were factors that raised the suspicion for malignancy for both general and orthopaedic surgery residents. Both groups were also appropriately wary of masses greater than 3.8 cm and were unlikely to resect larger masses without a confirmed diagnosis. In contrasting reality, tumors that are excised without appropriate preoperative planning and diagnosis and are subsequently referred for reexcision range anywhere from 5 cm to greater than 10 cm [5–7]. STS are classically taught as being deep and large [8], and while this may save larger, deep tumors from improper treatment, this excludes teaching about STS that are superficial and small and that they are frequently misdiagnosed and excised without proper preoperative planning. STS that are often improperly excised and are subsequently reexcised have a greater proportion of tumors that are superficial and small as compared to tumors that are excised with a definitive diagnosis prior to excision—a point that deserves adequate educational representation [6, 9]. 

If residents graduating from general surgery and orthopaedic surgery are able to be appropriately suspicious of questionable soft tissue masses, then why do we still see one-third of all STS mismanaged? Perhaps, the providers responsible for referring (i.e., general surgeons, general orthopaedic surgeons, plastic surgeons, etc.) such high numbers of patients with incompletely excised STS may not be those who might have recently finished their residency training but those who are far removed from it. Currently there are around 1000 graduates of general surgery [10, 11] and 650 in orthopaedic surgery per year [12]. This is in contrast to a total of 135,854 active surgeons in the US today [13]. Although we would hope to see a dilutional effect over time, we have not. This may possibly explain why despite currently adequately preparing surgeons in training, high rates of inappropriate STS excisions continue to exist. Educational efforts focused on practicing surgeons may be more beneficial to curtail the persistently high rates. 

This study also shows a lack of confidence among surgical residents in proper biopsy techniques of soft tissue masses. Mankin et al. had previously reported on the hazards of biopsy and deleterious effects of an improper biopsy by surgeons in practice [14]. Thus, it is imperative that residency programs incorporate proper biopsy techniques as an important objective. In particular, it is crucial that graduating residents are proficient and comfortable with performing proper biopsies techniques (i.e., avoiding transverse incisions, meticulous hemostasis, and narrow suture placement) [15, 16]. It is also equally important to teach physicians in training about the importance and significance of a biopsy and in turn a diagnosis prior to excision of unknown soft tissue masses. If the initial patient presentation is highly indicative of malignancy, the physician might refer the patient directly to a sarcoma center prior to initiating any diagnostic workup. However, for atypical masses, especially those that are small and superficial, a biopsy-proven diagnosis is of absolute necessity prior to resection by the nonsarcoma specialist. 

This survey relied on residents' interpretation of three-dimensional images. Oftentimes, an inaccurate radiology report guides patient treatment. Interpreting radiological imaging and reports is also a key tool in accurately diagnosing STS. Imaging has been shown to be crucial in diagnosing STS especially in its early course [17–19]. By correlating known surgical anatomy and clinical knowledge with radiological imaging, strength and validity can be added to radiology reports of suspicious soft tissue masses; following imaging reports without personally reviewing the images can sometimes steer a surgeon towards the dangerous path of inappropriately excising a STS. 

Factors that residents thought that they were not in their control and could influence a diagnosis included the role of pathology and a biopsy. The limited availability of pathologists and the subsequent complication in logistically coordinating biopsies were also factors that were reported as being impediments. As part of the education process, these limitations should not be used to diminish the role of biopsies and directly proceed to excision of the mass. Training should include education to overcome obstacles in performing biopsies, including a pathology referral or the option to refer the patients to specialist centers where they can be performed. In addition, specialist centers also enable the synthesis of clinical, pathological, and imaging data [20] from various specialties and allow for the creation of a structured plan for definitive excision. 

Residency training provides the time and framework for physicians to master proper techniques and become proficient in their field. Our study shows that the current generation of residents is able to appropriately be suspicious of possible malignancy in soft tissue lesions with increasing accuracy as they progress through training. Educational efforts in sarcoma care with an emphasis on the hazards of misdiagnosis should be implemented beyond the years of residency training and for physicians currently in practice.

## Figures and Tables

**Table 1:** Resident's characteristics of both general surgery and orthopaedic surgery groups.

	General surgery (*N* = 134)	Orthopaedics (*N* = 247)	*P* value
Age	30.5 (3.1)	30.1 (3.8)	.31
Gender (*N*, %)			.002
Female	87 (64.9)	51 (20.6)	
Male	47 (35.1)	196 (79.4)	
Years of training	3.0 (1.5)	3.1 (1.5)	.51
Dedicated STS rotation (*N*, %)			<.001
Yes	48 (35.8)	208 (84.2)	
No	86 (64.2)	39 (15.8)	
Dedicated STS teaching component			<.001
Yes	90 (67.2)	240 (97.2)	
No	44 (32.8)	7 (2.8)	

Continuous data presented as mean (±SD).

**Table 2:** Vignette responses by general surgery and orthopaedics residents.

Vignette	General Surgery		Orthopaedics		*P* value
	Benign	Malignant	Benign	Malignant	
1 (25 yo)	51 (38.1)	83 (61.9)	112 (45.3)	135 (54.7)	.17
2 (55 yo)	75 (56.0)	59 (44.0)	139 (56.3)	108 (43.7)	.95
3 (75 yo)	88 (65.7)	46 (34.3)	143 (57.9)	104 (42.1)	.14
4 (35 yo)	33 (24.6)	101 (75.4)	81 (32.8)	166 (67.2)	.10
5 (69 yo)	49 (35.6)	85 (63.4)	64 (25.9)	183 (74.1)	.03

Percentage of correct answers *M* (SD)	55.8 (.19)		56.4 (.21)		.81

Data presented as *N*, %. Pearson's Chi-square test.

**Table 3:** Residents' rating of characteristics that increase suspicion for malignancy.

	General surgery	Orthopaedics	*P* value
Factors raising suspicion for malignancy			
Largest size tumor that you would be comfortable with excising without a biopsy	3.8 (1.7)	3.8 (2.1)	.67
Rate the characteristic that increases suspicion for malignancy*			
Depth of the mass	4.9 (0.9)	5.2 (0.9)	<.001
Rate of growth	5.3 (1.0)	5.6 (0.6)	<.001
Imaging characteristics	5.4 (1.0)	5.6 (0.6)	<.001
Location of the mass	4.9 (0.9)	4.9 (1.0)	.55
Mobility of the mass	4.5 (1.1)	4.4 (1.1)	.24
Age of the patient	4.4 (1.1)	4.6 (1.0)	.01

All data presented as mean (±SD). Options for tumor size were presented as integers ranging from 1 cm to 10 cm.

*Note: scores on the scale ranged from 1 to 6, with 1 indicating “Strongly Disagree” and 6 indicating “Strongly Agree.”

**Table 4:** Rating of factors that impede a resident's decision to perform a biopsy prior to excision.

	General surgery	Orthopaedics	*P* value
Factors impeding decision to perform a biopsy*			
I have decreased access to persons performing needle biopsy	2.6 (1.5)	2.7 (1.5)	.43
I do not want to inconvenience the pathologist	1.6 (0.9)	1.5 (0.7)	.87
I have a lack of confidence in pathology to make a diagnosis on a FNA/core biopsy	2.5 (1.3)	3.0 (1.4)	<.001
I feel that whole specimen will lead to a more accurate diagnosis	3.5 (1.3)	3.7 (1.4)	.12
I feel that the likelihood of a soft tissue mass being malignant is low	3.2 (1.3)	2.8 (1.2)	<.001
I have concerns about the risks associated with biopsies	2.7 (1.4)	2.8 (1.3)	.42
I am not extensively trained on the use of biopsies in residency or fellowship	2.9 (1.5)	3.2 (1.5)	.02

All data presented as mean (±SD).

*Note: scores on the scale ranged from 1 to 6, with 1 indicating “Strongly Disagree” and 6 indicating “Strongly Agree.”

**Table 5:** Bivariate analyses for selecting correct clinical decision for general surgery residents.

Variable	OR (95% CI)	*P* value
Factors raising suspicion for malignancy		
Largest size tumor that you would be comfortable with excising without a biopsy	1.04 (0.95, 1.13)	.45
Depth of the mass	1.07 (0.90, 1.27)	.48
Rate of growth	0.89 (0.75, 1.04)	.15
Imaging characteristics	0.88 (0.73, 1.05)	.16
Location of the mass	0.96 (0.81, 1.15)	.67
Mobility of the mass	0.91 (0.79, 1.05)	.19
Age of the patient	0.91 (0.79, 1.05)	.19
Factors impeding decision to perform a biopsy		
I have decreased access to persons performing needle biopsy	1.02 (0.92, 1.13)	.74
I do not want to inconvenience the pathologist	1.03 (0.87, 1.21)	.77
I have a lack of confidence in pathology to make a diagnosis on a FNA/core biopsy	0.93 (0.83, 1.05)	.23
I feel that whole specimen will lead to a more accurate diagnosis	0.94 (0.84, 1.06)	.35
I feel that the likelihood of a soft tissue mass being malignant is low	0.94 (0.83, 1.05)	.28
I have concerns about the risks associated with biopsies	0.97 (0.86, 1.08)	.57
I am not extensively trained on the use of biopsies in residency or fellowship	0.96 (0.86, 1.06)	.40
Resident characteristics		
Age	1.03 (0.98, 1.08)	.30
Gender	1.12 (0.81, 1.55)	.49
How many years of training have you completed?	1.11 (1.00, 1.23)	.04*
Dedicated STS rotation	0.96 (0.70, 1.32)	.81
Dedicated STS teaching component	0.85 (0.61, 1.17)	.32

CI: confidence interval. Significance denoted by *P < .05.

**Table 6:** Bivariate analyses for selecting correct clinical decision for orthopaedic surgery residents.

Variable	OR (95% CI)	*P* value
Factors raising suspicion for malignancy		
Largest size tumor that you would be comfortable with excising without a biopsy	0.94 (0.89, 0.99)	.02*
Depth of the mass	1.05 (0.93, 1.18)	.44
Rate of growth	1.14 (0.95, 1.36)	.15
Imaging characteristics	1.00 (0.83, 1.22)	.97
Location of the mass	1.02 (0.91, 1.14)	.72
Mobility of the mass	0.91 (0.82, 1.01)	.08
Age of the patient	0.86 (0.77, 0.96)	.006*
Factors impeding decision to perform a biopsy		
I have decreased access to persons performing needle biopsy	0.98 (0.91, 1.06)	.69
I do not want to inconvenience the pathologist	0.84 (0.72, 0.98)	.03*
I have a lack of confidence in pathology to make a diagnosis on a FNA/core biopsy	1.00 (0.92, 1.08)	.91
I feel that whole specimen will lead to a more accurate diagnosis	0.95 (0.87, 1.03)	.19
I feel that the likelihood of a soft tissue mass being malignant is low	0.91 (0.83, 1.00)	.05
I have concerns about the risks associated with biopsies	0.91 (0.86, 1.03)	.18
I am not extensively trained on the use of biopsies in residency or fellowship	0.96 (0.89, 1.04)	.34
Resident characteristics		
Age	1.00 (0.97, 1.03)	.94
Gender	0.98 (0.74, 1.30)	.90
How many years of training have you completed?	1.10 (1.02, 1.19)	.01*
Dedicated STS rotation	0.92 (0.67, 1.25)	.58
Dedicated STS teaching component	0.72 (0.37, 1.14)	.34

CI: confidence interval. Significance denoted by *P < .05.
